# The Role of Selected Adipocytokines in Ovarian Cancer and Endometrial Cancer

**DOI:** 10.3390/cells12081118

**Published:** 2023-04-09

**Authors:** Sebastian Stępień, Paweł Olczyk, Joanna Gola, Katarzyna Komosińska-Vassev, Aleksandra Mielczarek-Palacz

**Affiliations:** 1 Department of Immunology and Serology, Faculty of Pharmaceutical Sciences in Sosnowiec, Medical University of Silesia in Katowice, 41-200 Sosnowiec, Poland; 2 Department of Community Pharmacy, Faculty of Pharmaceutical Sciences in Sosnowiec, Medical University of Silesia in Katowice, 41-200 Sosnowiec, Poland; 3 Department of Molecular Biology, Faculty of Pharmaceutical Sciences in Sosnowiec, Medical University of Silesia in Katowice, 41-200 Sosnowiec, Poland; 4 Department of Clinical Chemistry and Laboratory Diagnostics, Faculty of Pharmaceutical Sciences in Sosnowiec, Medical University of Silesia in Katowice, 41-200 Sosnowiec, Poland

**Keywords:** adipocytokines, ovarian cancer, endometrial cancer, obesity, inflammation

## Abstract

Due to their multidirectional influence, adipocytokines are currently the subject of numerous intensive studies. Significant impact applies to many processes, both physiological and pathological. Moreover, the role of adipocytokines in carcinogenesis seems particularly interesting and not fully understood. For this reason, ongoing research focuses on the role of these compounds in the network of interactions in the tumor microenvironment. Particular attention should be drawn to cancers that remain challenging for modern gynecological oncology—ovarian and endometrial cancer. This paper presents the role of selected adipocytokines, including leptin, adiponectin, visfatin, resistin, apelin, chemerin, omentin and vaspin in cancer, with a particular focus on ovarian and endometrial cancer, and their potential clinical relevance.

## 1. Ovarian Cancer and Endometrial Cancer

### 1.1. Ovarian Cancer

**Figure 1:**
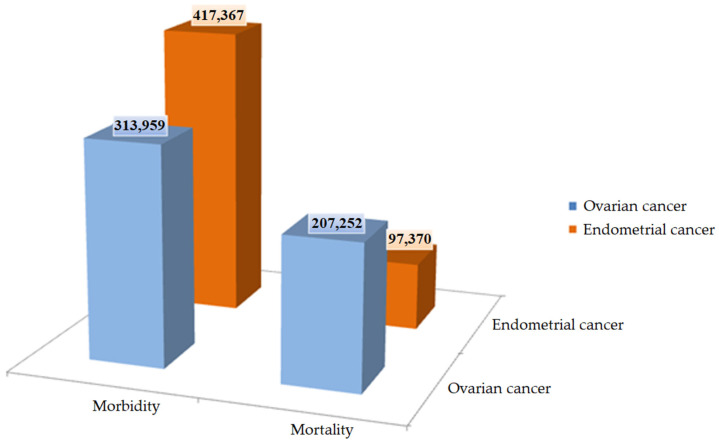
Epidemiology of ovarian cancer and endometrial cancer worldwide in 2020 [12].

Ovarian cancer (OC) is among the most dangerous gynecologic cancers, as it is associated with a high mortality rate [1,2,3,4,5,6,7,8,9,10]. In the general population, the risk of ovarian cancer averages 1.3% [3,11]. According to GLOBOCAN data, in 2020, there were 313,959 new cases of this cancer registered and 207,252 deaths attributed to ovarian cancer, as shown in Figure 1. These data allow us to conclude that ovarian cancer is the seventh most common cancer and the fifth most common cause of cancer-related deaths among patients worldwide [12]. U.S. and UK medical registries indicate that 1 in 6 women die from the development of ovarian cancer within the first 90 days of diagnosis [2]. The high mortality rate is associated with the diagnosis of ovarian cancer at a late stage of clinical progression. More than 70% of ovarian cancer cases are estimated to be diagnosed at clinical stage III or IV according to the FIGO (International Federation of Gynecology and Obstetrics) classification [13,14,15]. A stage I ovarian cancer diagnosis affects a good prognosis for patients, with a survival rate of about 90%, while stage II has a 70% survival rate. Unfortunately, the survival rate for stages III and IV is low, at less than 30%. In addition, it is presumed that 70% of patients will develop ovarian cancer recurrences, where the survival rate is low [3,6,7,8,9,10,13,14,16]. Late diagnosis of ovarian cancer is often associated with an asymptomatic course. Symptoms present in patients, such as abdominal pain, bloating, weight gain, back pain, incontinence, and irregular menstruation, are usually mistakenly associated with symptoms originating from the gastrointestinal tract or urinary tract, which delays the diagnosis of this disease [17,18,19,20]. Despite numerous studies conducted for many years, specific markers for early diagnosis of ovarian cancer have not yet been developed [9,10,19,21,22,23,24].

### 1.2. Endometrial Cancer

Endometrial cancer (EC) is among the most common gynecological cancers in developed countries. Unfortunately, the incidence of this cancer is increasing worldwide, and this trend is expected to continue for the next 10 years [25,26,27,28,29,30,31,32]. Based on GLOBOCAN statistics, 417,367 new cases were diagnosed in 2020, and 97,370 deaths were registered, as presented in Figure 1. The above data makes EC the sixth most common cancer among women [12,33]. The incidence of endometrial cancer is estimated at nearly 5.9% [28]. Endometrial cancer is diagnosed primarily in postmenopausal women. It is estimated that the average age of women diagnosed with EC is 60 years. In contrast, 14% of EC cases involve women under 45. According to recent reports, 70% of pre-menopausal endometrial cancer cases involve patients who are classified as non-breastfeeding women. Pregnancy and childbirth have a protective effect on the development of EC [34,35,36]. The prognosis for patients and the 5-year overall survival rate depends on the stage of the disease according to the FIGO classification; the higher the clinical stage, the lower the prognosis and survival rate. It is reported that for stage I, the overall survival is 80–90%, for stage II—80%, for stage III—50–70%, while for stage IV it is about 20% [36,37]. In the case of endometrial cancer, the diagnosis is made at an early stage of the disease, as the development of EC is characterized by specific symptoms that are already apparent at the early stages of the disease [25,32,38].

According to recent data, the main factor in developing endometrial cancer, as in ovarian cancer, is obesity, which is found in more than 57% of cases. It has been shown that women with a normal BMI (Body Mass Index) have a low risk of developing endometrial cancer of about 3%. Moreover, as BMI increases by another 5 units, the risk of EC recurrence elevates by as much as 50% [34]. Estrogen is believed to be a key compound contributing to the development of endometrial cancer. In premenopausal women, ovaries’ cyclic estrogen expression associated with the physiological menstrual cycle stimulates endometrial proliferation. In postmenopausal women, on the other hand, adipose tissue, in particular, is a source of estrogen. Preadipocytes, adipocytes and mesenchymal stem cells of adipose tissue become a source of aromatase, an enzyme necessary for converting androgens into estrogens. It has been observed that with age and degree of obesity, aromatase concentration and activity increase, which may contribute to uncontrolled endometrial proliferation [34,39]. On the other hand, obesity is associated with ongoing chronic inflammation in the body, which alters adipose tissue metabolism and leads to increased secretion of many compounds, especially hormones, adipokines, inflammatory cytokines, growth factors and many enzymes. The numerous factors thus secreted interact endocrinally, autocrinally and paracrinally with physiological cells and tissues, contributing to the initiation of the process of carcinogenesis [40,41,42,43].

## 2. Adipocytokines

The body’s adipose tissue plays an important role in its functioning by performing endocrine, metabolic and immunoregulatory functions [44]. It has been shown to produce approximately 600 different pro- and anti-inflammatory signaling mediators, including leptin, resistin, adiponectin, visfatin and apelin—referred to as adipocytokines [44,45,46,47,48]. Adipocytokines are biologically active molecules that are mainly produced by adipocytes. Physiologically, they are involved in regulating the processes of appetite and satiety and maintaining energy balance by affecting lipid metabolism, maintaining adequate glucose levels, blood pressure, insulin sensitivity, condition inter-tissue exchange throughout the body, and thus play an essential function in maintaining endocrine homeostasis [46,49,50,51,52]. Recent studies show that adipocytokines, interacting through endocrine, paracrine and autocrine pathways, significantly affect the processes of carcinogenesis of malignancies, mainly breast cancer, ovarian cancer and uterine cancer, increasing the risk of developing these cancers, especially in postmenopausal women [49,53,54]. In addition, some adipocytokines, such as adiponectin, chemerin and omentin, may also exhibit anticancer effects [44,46].

## 3. The Role of Obesity in the Development of Cancer

Obesity is now recognized as a chronic disease that has long had an increasing incidence worldwide [55]. Overweight (BMI > 25 kg/m^2^) and obesity (BMI > 30 kg/m^2^) in adults (over 18 years of age) are thought to occur at a frequency of 39% and 13%, respectively [56]. According to data published by the International Agency for Research on Cancer (IARC), being overweight and especially obese is strongly associated with an increased incidence of thirteen cancers, most notably ovarian and endometrial cancer. It is associated with a higher risk of cancer death [56,57,58]. The burden of cancer occurrence correlated with obesity is 11.9% in the male population and 13.1% in the female population worldwide [59].

Obesity modifies adipose tissue metabolism and leads to increased secretion of mainly adipocytokines, pro-inflammatory cytokines, growth factors and hormones. Disruption of homeostasis between these molecules results in the initiation of chronic inflammation in the body, characterized by the activation of immune cells, the elevation of local and systemic inflammatory cytokines and subsequent deregulation of immune system mechanisms [40]. In addition, in the excessive adipose tissue of obese individuals, there is an accumulation of immune cells, which mainly include neutrophils, mast cells, T and B lymphocytes and NK cells, which in turn contribute to the maintenance of inflammation. In addition, the development of inflammation is stimulated by numerous cytokines, mainly IL-1, IL-6 and TNF-α. The interplay of these mechanisms and the increased endocrine activity of the altered adipose tissue contribute to the proliferation, invasion and metastasis of tumor cells [40,60,61,62].

Further mechanisms linking obesity to the process of carcinogenesis include genetic disorders. The available data indicate the involvement of obesity in disrupting the mechanisms of DNA damage and repair, and thus excessive obesity may be linked to the process of genetic instability [63]. In obesity, oxidative stress and lipid dysregulation lead to excessive reactive oxygen species (ROS) generation, and these, in turn, can induce oxidative DNA damage. Moreover, such lesions as single-strand breaks (SSB) or double-strand breaks (DSB) are present in the lymphocytes of obese patients [64]. An additional issue is the ineffectiveness of DNA repair systems, which can lead to genetic instability [63]. It is well known that in endometrial and ovarian cancer, DNA repair systems are disrupted. The most common defect in ovarian cancer concerns the homologous recombination (HR) DNA repair system [65], while impairment of the DNA mismatch repair (MMR) system is more frequent in endometrial cancer [66]. Obesity influenced DSB repair systems and nucleotide excision repair (NER) [64]. High-fat diet affects the epigenetic regulation of genes encoding proteins involved in repair systems and induces chemical modifications of their products, leading to impairment of DNA repair [64]. Additionally, an imbalance in pro- and anti-inflammatory adipocytokines secretion in obesity leads to the activation of several oncogenic signaling pathways, including NFκB involved in the modulation of DNA repair in cancer [64]. It was found that adiponectin induces activation of peroxisome proliferator-activated receptor-α (PPARα) [67]. PPARα acts as a nuclear transcription factor involved, among others, in the activation of Chek1-related pathways responsible for cell cycle regulation and induction of HR DNA repair [68]. Visfatin (NAMPT) is the rate-limiting enzyme in NAD+ biosynthesis, and its expression is excessive in many cancers [69]. NAD+ is a substrate for PARP (poly(ADP-ribose)polymerase)1, PARP2 and PARP325 involved in the DNA damage recognition and induction of DNA repair systems. Moreover, Zhu et al. [70] have demonstrated that visfatin suppresses HR while promoting NHEJ (non-homologous end joining) DNA repair, resulting in genomic instability.

Numerous studies conducted show that obesity is a major factor in the development of many cancers, including ovarian and endometrial cancer [71,72,73,74,75,76].

Assidi et al. [77] indicate that a 5-unit increase in BMI is associated with a 6% increase in the incidence of ovarian cancer. Moreover, women struggling with obesity who develop ovarian cancer can be characterized by the survival rate compared to women diagnosed with ovarian cancer and normal weight. In contrast, Crean-Tate et al. [73] report that women with obesity of childbearing age have twice the risk of developing ovarian cancer compared to women with a normal BMI, and the likelihood of recurrence and death in women with obesity is increased by as much as 40%. On the other hand, Dixon et al. [78] indicate that higher body fat mass positively correlates with the risk of low-grade serous ovarian cancer.

Shaw et al. [43] report that women with a BMI in the range of 30–35 kg/m^2^ have a 2.6-fold increased risk of developing endometrial cancer, while women with severe obesity BMI > 35 kg/m^2^ have a 4.7-fold increased risk of endometrial cancer compared to women of normal weight (BMI < 25 kg/m^2^). According to the World Cancer Research Fund (WCRF), an increase in BMI by 5 units increases the risk of developing endometrial cancer by as much as 50% [79]. Kokts et al. [74] indicate that at the time of endometrial cancer diagnosis, obesity correlated with an increased risk of cancer recurrence and mortality, which the authors reported was not directly related to cancer development. Interestingly, Kho et al. [80] showed that lipid fractions circulating in the blood are associated with an increased risk of endometrial cancer. The authors showed that low-density cholesterol (LDL) and elevated high-density cholesterol (HDL) are likely risk factors for the development of endometrial cancer. Among many associated risk factors, metabolic syndrome comprises central adiposity, hyperglycemia, arterial hypertension, and atherogenic dyslipidemia, which is an important one. Chronic adipose tissue inflammation and insulin resistance constitute the ideal environment for developing gynecological cancer. Moreover, obesity promotes insulin resistance, hyperinsulinemia and hyperglycemia, which increases gynecological cancer risk and is related to poor prognosis [39,41,44,80].

## 4. Leptin

As early as 70 years ago, mouse fertility researchers noted that there is a factor that affects adipose tissue causing the onset of obesity. Despite many studies conducted, it was only found that this factor was derived from adipose tissue, but it could not be isolated [81]. A pioneering achievement was made by Zhang et al. [82], who in 1994 identified and cloned the factor, which was later named “leptin” from the Greek word leptos meaning thin [83,84].

Leptin is the product of the *LEP* gene, located on chromosome 7. It is a peptide consisting of 167 amino acids, occurring as a tertiary protein structure, and has a molecular weight of 16 kDa [83,85,86,87,88]. While leptin is synthesized by adipocytes of adipose tissue, current reports indicate that leptin is also present in other tissues, including the placenta, mammary gland, ovary, skeletal muscle, stomach, and pituitary gland [85]. The physiological serum leptin concentration is 16 ng/mL, while the reference value range is 10–20 ng/mL [81,89].

The physiological role of leptin is to regulate food intake and maintain energy homeostasis in the body [90,91].

At the hunger and satiety center in the hypothalamus, leptin inhibits neuropeptide Y (NPY) and Agouti-related protein (AgRP). In addition, it stimulates αMSH (α-Melanocyte Stimulating Hormone) by stimulating POMC (Proopiomelanocortin), resulting in reduced food intake. Furthermore, leptin initiates cancer progression by affecting the initiation of signal transducers and activator of transcription 3 (STAT-3) and STAT-3 terminal gene expression resulting in melanocortin inhibition, stimulation of signal transducers and activator of transcription 5 (STAT-5), leading to altered gene expression, and stimulation of PI3K (Phosphatidylinositol-3-kinase), which interacts with insulin receptor pathways [88,92,93,94].

Leptin induces rapid changes in body temperature without altering energy expenditure. In addition, leptin administration results in reduced heat loss, presumably through vasodilation. Numerous studies in ob/ob mice indicate that a reduction in leptin concentration does not result in an inability to protect against lower temperatures, only to change the thresholds for stimulation of thermoregulatory effectors [95,96,97,98,99,100].

This regulation is made possible by the existence of receptors for leptin, which are present throughout the body. Leptin receptors have been classified as class I cytokine receptors. The action of leptin is possible through the specific trans-membrane receptor LEP-R, located in the central nervous system and exists in the form of six isoforms that share a common leptin-binding domain but differ in the length of the cytoplasmic regions. Considering this criterion, the isoforms were labeled LEP-Ra, LEP-Rb, LEP-Rc, LEP-Rd, LEP-Re and LEP-Rf, respectively. In addition, they were divided into three classes: short isoforms, long isoforms and secreted isoforms [81,83,85,89,90,91].

**Figure 2:**
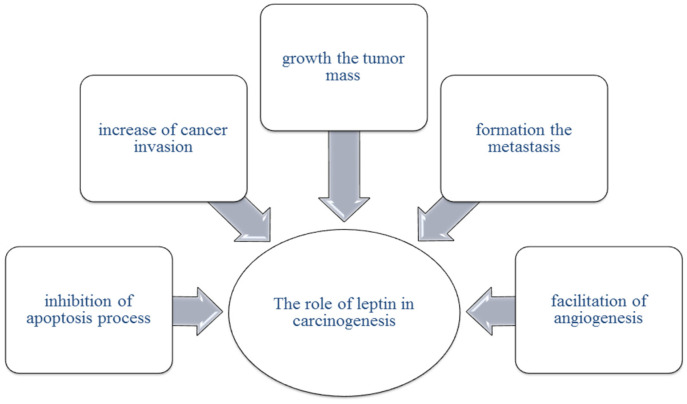
Potential role of leptin in carcinogenesis [101,107,108,109,110].

In addition to its physiological role in the body, leptin affects cancer development. Recent studies report that elevated leptin levels correlate with an increased risk of certain malignancies, which mainly include renal cell carcinoma [101], intestinal cancer [102], pancreatic cancer [103], breast cancer [104] and gynecological cancers, including endometrial cancer [105] and ovarian cancer [106]. The potential role of leptin in carcinogenesis is shown in Figure 2.

### 4.1. The Role of Leptin in Ovarian Cancer

To date, research has been ongoing into the direct effects of leptin on the development and progression of ovarian cancer. More than 10 years ago, Uddin et al. [111] used cell cultures of SKOV-3 and MDAH2774 lines to prove the above theory’s validity. They treated ovarian cancer cells of both lines with different leptin concentrations, ranging from 20–200 ng/mL. The results thus obtained allowed them to conclude that this adipocytokine significantly stimulated the proliferation of ovarian cancer cells. Moreover, the authors found that cell growth was dependent on the concentration of leptin in the medium. A similar analysis was performed later by Chin et al. [93], who used two ovarian cancer cell lines: SKOV-3 and OVCAR-3. Cells of both lines were also stimulated with leptin at different concentrations (1 μM, 10 μM and 100 μM). The researchers obtained similar results to previous ones, which indicate that leptin stimulates the proliferation of ovarian cancer cells and the growth of ovarian cancer cells correlated with higher leptin concentrations in the medium.

Uddin et al. [111] used plate-seeded ovarian cancer cells of the SKOV-3 and MDAH2774 lines and, after a specified period, added leptin-containing medium at a concentration of 100 ng/mL to a part of the plates as a test sample and added leptin-free medium to the other part of the plates as a control. It was observed that stimulating OC cells with leptin significantly reduced apoptosis. The results demonstrate leptin’s inhibitory role in the apoptosis process in ovarian cancer cells, and therefore leptin can be considered an antiapoptotic factor in tumorigenesis.

Matte et al. [112] first correlated the concentration of the marker CA125 (Carbohydrate Antigen 125) in serum and ascites and leptin concentration in ascites in patients diagnosed with High-Grade Serous Ovarian Carcinoma (HGSOC) with baseline clinical resistance to standard first-line therapy, which includes platinum-based treatment. During the analysis, they observed that high CA125 and leptin ascites ratios correlate with shorter overall survival (OS) and low progression-free survival (PFS). According to the authors, the ratio of CA125 and leptin may be useful biomarkers for diagnosing ovarian cancer, but this requires further analysis.

Leptin has been implicated in regulating various malignant phenotypes of ovarian cancer through its involvement in the hypothalamic-pituitary-gonadal axis and multiple signaling pathways. Thus, this axis is associated with hormone receptors and hormone-specific receptors [106,113]. Previous studies indicate that FSH (Follicle Stimulating Hormone), unlike LH (Luteinizing Hormone), promotes the growth and development of cancer cells and limits the process of cancer cell apoptosis in ovarian cancer. In a mouse model, increased leptin levels significantly increased serum FSH and TSH (Thyroid Stimulating Hormone) levels, while increased leptin levels decreased serum LH levels [93]. In contrast, other reports indicate that leptin can regulate tumor proliferation and invasion via signaling pathways. For example, leptin stimulates the activation of MMP7 (Matrix Metallopeptidase 7), causing increased signaling of ERK (Extracellular Signal Regulated Protein Kinase) and JNK (c-Jun N-Terminal Kinase) pathways, resulting in tumor invasion. Similar effects are induced by leptin-stimulated urokinase-type plasminogen activator (uPA) expression, where this signaling is mediated by the RhoA-ROCK (Ras homolog gene family, member A (RhoA)-effector Rho-associated protein kinase) pathway [109].

Gu et al. [106] in their study analyzed the role of leptin in ovarian cancer patients undergoing chemotherapy treatment. They analyzed survival rates in 1656 ovarian cancer patients treated with various chemotherapy programs and found that high leptin level correlates with decreased survival rates. They also showed that leptin enhances the chemoresistance of ovarian cancer to treatment with platinum in combination with PTX/TXT (Paclitaxel/Docetaxel). The authors concluded that high leptin levels do not inhibit tumor cell division at the G2/M stage by blocking the inhibitory effect. In addition, Kukla et al. [114] report that in patients treated with platinum compounds in combination with paclitaxel/docetaxel, leptin has an adverse effect on prognosis.

### 4.2. The Role of Leptin in Endometrial Cancer

Recent studies show that elevated leptin levels are associated with the degree of myometrial infiltration, the occurrence of secondary tumor foci in lymph nodes also associated with lymphatic vessel involvement, and a low survival rate [41]. The molecular mechanism of endometrial cancer development is associated with overexpression of the leptin gene and the receptor for leptin (ObR), as well as HIF-1 (Hypoxia Inducible Factor), which lead directly to the activation of STAT family proteins responsible for the development of inflammation, proliferation, formation of secondary tumor foci and chemo-resistance [30]. In endometrial cancer, leptin directly affects the activation of STAT3 proteins contributing to the immune escape mechanism.

## 5. Adiponectin

Adiponectin (APN) was discovered in 1995 as the so-called Adipose Complement Related Protein of 30 kDa (Acrp30), where it was subsequently named adiponectin. Adiponectin is expressed in adipose tissue and well-differentiated cells in cell culture. Mouse adiponectin is a polypeptide encoded by 247 amino acids. There is a signaling sequence on the N-terminal fragment, followed by a hyper-variable region, as well as a collagen domain that exhibits a 22-fold GXY repeat and a globular domain [115,116,117]. Considering the protein plane, the globular C-terminal domain of adiponectin shows a great similarity to complement factor C1q subunits. Researchers indicate that the adiponectin and C1q families may be derived from a single basic parent molecule [118,119]. A year later, in 1996, scientists compiling a cDNA (Complementary Deoxyribonucleic Acid) base of human adipose tissue described a protein, calling it adipose most abundant gene transcript 1 (apM1)—adiponectin. Human adiponectin is produced by white adipose tissue and comprises 224 amino acids. The gene for this protein is located on chromosome 3q27. Adiponectin is a multimeric protein that exists in several biologically active isoforms. Transcription and translation processes produce low-molecular-weight LMW-Ad trimers (90 kDa), medium-molecular-weight MMW-Ad hexamers (180 kDa) and high-molecular-weight HMW-Ad dodecamers to octadecamers (360 and 400 kDa). HMW-Ad predominates in plasma [108,118,120,121,122,123].

Adiponectin is a protein produced by white adipose tissue. Recent studies indicate that adiponectin can also be synthesized and secreted in other organs, which mainly include the brain, retina, salivary glands, liver, colon or placenta [62,124]. The serum concentration of adiponectin in healthy individuals is 3–30 μg/mL [62,120]. In contrast, in obese individuals, adiponectin concentration is closely related to adipose tissue and decreases with the severity of obesity [46]. It exhibits a sexual dimorphism manifested by concentrations more than twice as high in women as in men [118,125]. Such a ratio is already formed during perinatal life. Researchers suggest that reduced adiponectin concentrations in males are related to testosterone activity. Moreover, in adult men, testosterone (T) affects the processes of synthesis, complex formation or also degradation of adiponectin. Adiponectin is physiologically involved in glucose metabolism mainly by inhibiting the gluconeogenesis pathway in hepatocytes [125]. It exhibits anti-diabetic, anti-atherosclerotic and also anti-inflammatory effects. The anti-inflammatory effect is a result of combating inflammatory cytokine function and inhibition of TNF-α (Tumor Necrosis Factor Alpha) synthesis. It is achieved by blocking p38 mitogen-activated protein kinase (p38MAPK) and inflammatory signaling from macrophages mediated by TNF-α [119,121,123,126].

Adiponectin acts in the body through specific classical receptors, AdipoR1 and AdipoR2, as well as through a non-specific receptor—T-cadherin. AdipoR1 is most abundant in skeletal muscle and mucous membranes, while AdipoR2 is found in hepatocytes [62,123,127,128,129]. Structurally, AdipoR1 and AdipoR2 consist of seven transmembrane domains with reverse C-terminal and N-terminal localization compared to G protein-related receptors. Both of these receptors show 67% structural homology to each other but nevertheless have different affinities for each isoform [62].

**Figure 3:**
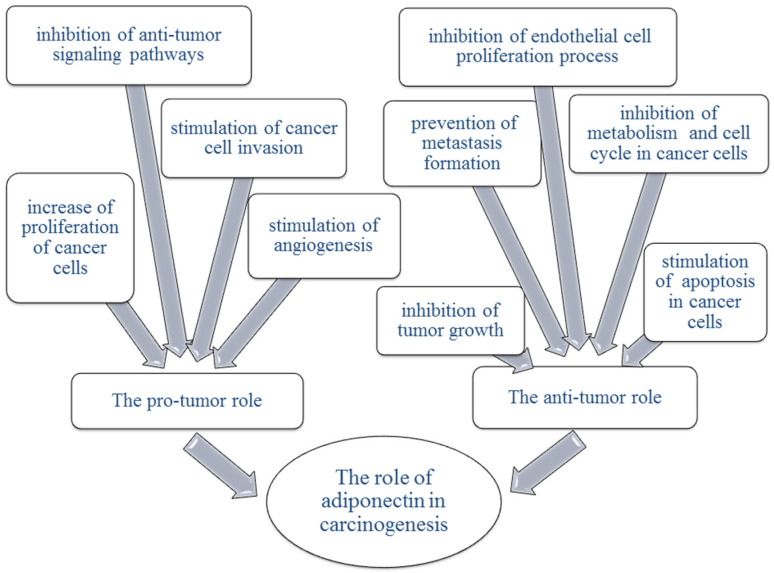
Potential role of adiponectin in carcinogenesis [44,62,108,119,127,130,131,132].

Adiponectin is also considered a key mediator in the development of obesity-related cancers [44,62,106]. The potential role of adiponectin in carcinogenesis is shown in Figure 3.

### 5.1. The Role of Adiponectin in Ovarian Cancer

Adiponectin has been linked as a factor preventing carcinogenesis due to its inhibition of angiogenesis, suppression of growth and proliferation, inhibition of invasion, migration, formation of cancer metastasis, and anti-inflammatory effects [62,108,127]. On the other hand, hypoadiponectinemia is a condition that facilitates tumorigenesis due to the initiation of inflammation by stimulating the synthesis of inflammatory mediators, which is known to play a key role in the initiation and progression of cancer, including ovarian cancer [133]. Additionally, the role of leptin and adiponectin in ovarian cancer has been linked. Diaz et al. [134] indicate that a high leptin/adiponectin ratio correlated with a poor prognosis for a patient diagnosed with ovarian cancer. On the other hand, Słomian et al. [135] found a correlation between leptin and adiponectin levels before treatment with a favorable response of the organism to the chemotherapy administered. It is now also believed that adiponectin receptors play an important role in carcinogenesis. Decreased expression of adiponectin receptors, mainly AdipoR1, is observed in epithelial ovarian cancer. Hence it is speculated that AdipoR1 may be a new prognostic factor for this cancer [62,114]. In their study, Hoffmann et al. [122] found that both AdipoR1 and AdipoR2 showed expression in multiple epithelial ovarian cancer cell lines and noted that expression was lower than in a granulosa tumor cell line (COV434). Li et al. [136] found a reduced expression of AdipoR1 in epithelial ovarian cancer compared to normal ovarian tissue. The authors also showed a correlation between AdipoR1 expression and the FIGO stage and the presence of ascites in patients with epithelial ovarian cancer (EOC). They concluded on the carcinogenic role of this receptor in the development of ovarian cancer.

### 5.2. The Role of Adiponectin in Endometrial Cancer

A study by Wang et al. [137] concludes that adiponectin levels are significantly reduced in patients with endometrial cancer compared to serum levels of this parameter in healthy women. It is believed that adiponectin levels <8 mg/dL are associated with increased cancer stage [41]. Analyzing the results obtained, the authors showed a negative correlation between BMI and serum adiponectin concentration, as well as between serum adiponectin concentration and the amount of fat mass in obese subjects, who were additionally found to have reduced concentrations of this parameter. According to the present data, the authors indicate a strong association between obesity and the risk of developing endometrial cancer [138]. Dashti et al. [139] were among the first to analyze the contribution of multiple parameters associated with obesity and endometrial cancer. They found that the interplay of adiponectin, inflammation, C-peptide and estrogen increases the risk of endometrial cancer as much as 70-fold in those diagnosed with obesity. On the other hand, elevated serum adiponectin levels can reduce the risk of endometrial cancer. According to researchers, each 5 μg/mL increase in serum adiponectin concentration results in an 18% reduction in the disease risk [137].

It is also known that not only reduced adiponectin concentrations affect the development of endometrial cancer. It has also been proven that reduced expression of AdipoR1 during the development of endometrial cancer is associated with an increase in the stage of endometrial cancer development and the occurrence of lymph node metastasis [131].

The results of a number of studies show that serum leptin and adiponectin levels are associated with an increase in the frequency of ovarian cancer in an inverse manner, i.e., increased leptin levels and decreased adiponectin levels have a pro-cancerogenic effect [114].

## 6. Visfatin

Visfatin was first cloned from lymphocytes in 1994. It was initially identified as a novel cytokine referred to as pre-B cell colony-enhancing factor (PBEF) due to the effect of IL-7 (Interleukin 7) on the formation of mouse pre-B cell colonies from early precursor B-lineage cells. Nowadays, visfatin is also known as nicotinamide phosphoribosyltransferase (NAMPT), as it can catalyze the synthesis reaction of nicotinamide mononucleotide (NMN) from nicotinamide (NAM) and 5-phosphoribosyl-1-pyrophosphate [54,140,141,142,143,144]. Visfatin is encoded by the *NAMPT* gene on the long arm of chromosome 7 (7q22) [145]. This novel adipokine comprises 163 amino acids and has a molecular weight of 52 kDa [144,146,147]. Human visfatin is believed to be produced primarily by adipocytes and adipose tissue macrophages [54]. In addition, increased expression of visfatin is shown in other tissues and organs, which mainly include bone marrow, liver, muscle, heart, lung and placenta, with the highest expression found in the liver and muscle [141]. Visfatin expression in tissues is regulated by cytokines, mainly TNF-α, IL-6 (Interleukin 6) and lipopolysaccharide (LPS) [142]. In addition, visfatin exhibits autocrine, paracrine and endocrine functions. In addition to its involvement in nicotinamide adenine dinucleotide (NAD) synthesis, it participates in the activation of the insulin signaling cascade, enhances glucose uptake and inhibits glucose release, promotes VEGF synthesis, and is involved in the regulation of many signaling pathways, mainly PI3K, Akt, ERK1/2, MAPK, or STAT3. 

**Figure 4:**
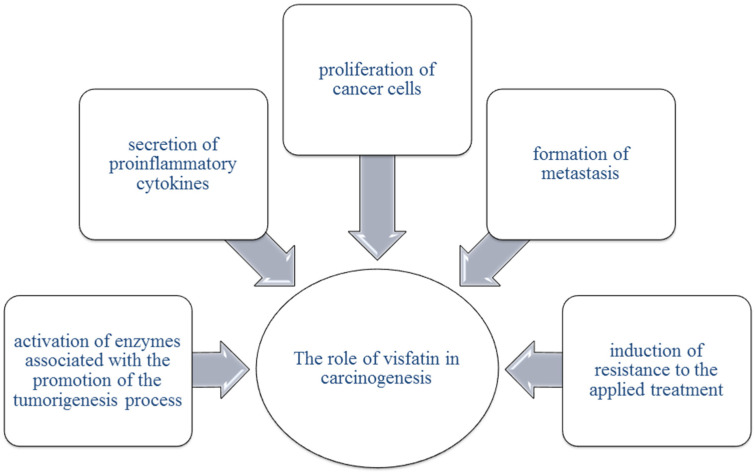
Potential role of visfatin in carcinogenesis [151,152,153,154].

Moreover, visfatin plays a regulatory role in some inflammatory diseases—dysregulation of the normal function of visfatin results in a number of pleiotropic or pathophysiological effects. Overexpression of visfatin in tumor tissue and its elevated serum levels have been found in patients with malignant tumors, suggesting the involvement of visfatin in carcinogenesis. Moreover, disruption of the NAD+ synthesis pathway and consequent increase in the NAD+/NADH ratio promotes the progression of many cancers [140,144,148,149,150]. The potential role of visfatin in carcinogenesis is shown in Figure 4.

### 6.1. Visfatin in Ovarian Cancer

Visfatin in ovarian cancer is mainly known as a potential therapeutic target. It is known that physiologically NAMPT plays an important role in the biosynthesis of NAD+, an essential molecule in cellular metabolism. Nevertheless, it has been demonstrated over recent years that NAMPT also plays an important role in the pathomechanism of cancer development, including ovarian cancer. Developing and growing cancer cells require significantly more NAD for their cellular metabolism compared to physiological cells. In addition, NAMPT-dependent NAD+ biosynthesis is required for the activation of NAD+-dependent enzymes, which include PARP1 (Poly (ADP-ribose) Polymerases-1), SIRT1 (Sirtuin 1), SIRT6 (Sirtuin 6) and CD38 (Cluster of Differentiation 38). All of these enzymes can promote carcinogenesis. The pro-cancer function is based on the increased expression of NAMPT, which is associated with the secretion of pro-proliferative cytokines, promoting the growth and development of cancer cells, stimulating angiogenesis, promoting metastasis or affecting genome stability [155,156,157,158,159,160,161,162].

Many available data indicate that reducing NAD+ in the tumor microenvironment is currently considered an optimistic concept for cancer therapy. Therefore, efforts on this topic aim to identify clinically useful NAMPT inhibitors. To date, several such compounds have been identified, such as—FK866 ((E)-N-[4-(1-benzoylpiperidin-4-yl)butyl]-3-(pyridin-3-yl)acrylamide), CHS828 ((E)-1-(6-(4-chlorophenoxy)hexyl)-2-cyano-3-(pyridin-4-yl)guanidine), GMX1777 (Teglarinad chloride), LSN3154567 (2-Hydroxy-2-methyl-N-[1,2,3,4-tetrahydro-2-[2-(3-pyridinyloxy)acetyl]-6-isoquinolinyl]-1-propanesulfonamide), A1293201((S)-N-(4-(((tetrahydrofuran-3-yl)methyl)carbamoyl)phenyl)isoindoline-2-carboxamide) and OT-82 (N-(3-(1H-pyrazol-4-yl)propyl)-3-((4-fluo-rophenyl)ethynyl)-4-(pyridin-4-yl)benzamide). The first known NAMPT inhibitor was FK866 [163,164,165,166]. As reported by Kudo et al. [167] and other researchers, the use of the FK866 inhibitor for treatment results in a decrease in the intensity of glycolysis and, thus, a decrease in cellular ATP (Adenosine Triphosphate) synthesis in the A2780 cell line, which limits the availability of energy for cellular metabolism [165,167]. On the other hand, the use of inhibitors enhances cancer cell apoptosis by activating caspase-3 and caspase-7, which was evident in increased lactate dehydrogenase (LDH) levels, a marker of tissue damage [167].

Whereas Nacarelli et al. [168] showed that NAMPT inhibition through inhibitors, particularly FK866 and GMX1778, affects CSCs (Cancer Stem Cells) associated with aging in ovarian cancer. As shown in a mouse model, inhibitor application slowed tumor growth, delayed relapse, and improved survival.

Li et al. [160] were the first to note a correlation between the presence of mutations in the *BRCA1* gene (Breast Cancer Associated Gene 1) and NAD synthesis. The authors report that there is a likely correlation between BRCA1 and NAD. It has been documented that overly intensive NAMPT-dependent NAD synthesis can activate BRCA1 transcription, while inactivation of BRCA1 stimulates NAD production. The mechanism is based on feedback.

### 6.2. Visfatin in Endometrial Cancer

Available data indicate that in the course of endometrial cancer, its average concentration is significantly higher than in healthy women [169,170,171,172]. Ilhan et al. [173] report that the average serum concentration of visfatin in patients with endometrial cancer was 14.9 ng/mL compared to the control group, where the average visfatin concentration was 8.1 ng/mL. The authors also indicate that concentrations of this adipocytokine >26.8 ng/mL are associated with an advanced degree of myometrial infiltration. During the development of endometrial cancer, visfatin exhibits anti-apoptotic, proliferative, and proangiogenic effects and promotes metastasis formation [41,169]. Moreover, elevated concentrations of visfatin lead to the stimulation of the cellular process in the G1/S phase, in consequence, causing inhibition of apoptosis in Ishikawa cells and KLE (a cell line that was isolated from the endometrium) [169]. Visfatin has also been shown to significantly affect the progression of malignant endometrial cancer via activation of the insulin receptor (IR) and PI3K/AKT and MAPK/ERK signaling pathways [174].

## 7. Resistin

Resistin was discovered in 2001 as a mouse protein that, as noted, was over-synthesized and secreted by adipose tissue in mouse models of genetic and dietary obesity. Its name, resistin, comes from the role it plays in insulin resistance. Resistin is encoded by the *RETN* gene on chromosome 19 (19p13.2). Resistin is a polypeptide consisting of 108 amino acids with a molecular weight of 12.5 kDa and containing numerous cysteine residues. It shows only 59% similarity in structure compared to its mouse counterpart, which may reflect the different functions it performs. Human resistin is most commonly found in two conformations—as a trimer (45 kDa) and as an oligomer (660 kDa). In serum, it occurs as a dimeric protein composed of two 92-amino acid polypeptides linked by a disulfide bridge and its physiological concentration ranges from 7 to 22 ng/mL. In humans, monocytes are the main source, while in rodents, it is mostly produced by adipocytes [175,176,177,178,179,180]. It has been shown that in obese individuals, the resistin concentration is above the upper limit of reference values [181].

The molecular mechanism of resistin’s action remains to be elucidated. The specific receptors for resistin are not known. It is thought that this adipokine may interact through Cyclase-Associated Protein 1 (CAP1), Toll-Like Receptor 4 (TLR4), Isoform of Decorin (ΔDCN) and tyrosine kinase-like orphan receptor 1 (ROR1). What is known, however, is that resistin activates Akt, MAPK, ERK1/2, STAT3 and PPARγ (Proliferator-Activated Receptor-gamma) in various tissues. Its autocrine, paracrine and endocrine effects on many cells and tissues have been demonstrated [176,178,182,183]. In addition, a positive correlation was found between inflammatory parameters such as CRP (C-Reactive Protein), TNF-α, and also IL-6 in the course of many diseases [183].

**Figure 5:**
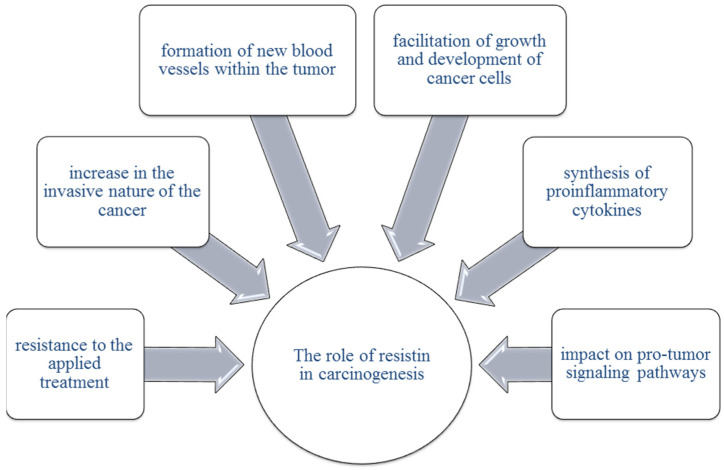
Potential role of resistin in carcinogenesis [53,54,94,178,184].

Current reports indicate that abnormal expression of resistin and its receptors are found in the course of many malignancies, particularly breast cancer, esophageal squamous cell carcinoma, gastric cancer, endometrial adenocarcinoma and ovarian cancer [183,184,185]. The potential role of resistin in carcinogenesis is shown in Figure 5.

### 7.1. Resistin in Ovarian Cancer

Resistin’s pathomechanism of ovarian cancer progression is based on modulatory effects of the immune system as well as the involvement of signaling pathways. Resistin stimulates the synthesis of TNF-α, IL-6 and IL-12 (Interleukin 12) in adipocytes and immune cells involved in ovarian cancer progression. It has also been found that cancer cells are capable of directly synthesizing these factors, resulting in in vitro expression of TNF-α and IL-6 1000-fold higher in cancer cells compared to physiological ovarian epithelial cells [184]. Studies by Pang et al. [53] indicate that resistin is capable of promoting and progressing ovarian cancer cells, which was associated with the growth of resistin-stimulated SKOV3 and CAOV3 lineage cells. The researchers also noted that cell growth was mediated by mTOR and P70S6K activation. In addition, it was shown that resistin positively correlates with tumor cell migration in ovarian cancer, contributing to ovarian cancer progression. Resistin in ovarian cancer interacts via the PI3K-Akt-Sp1 signaling pathway to stimulate the synthesis of MMP-2 and VEGF and lead to the formation of new blood vessels [184]. Qiu et al. [186], in their culture studies on A2780 and SKOV-3 lines, confirm resistin’s involvement in VGF and MMP-2 factor-mediated angiogenesis. In addition, resistin influences the generation of the invasive character of ovarian cancer cells by inhibiting miRNAs let-7, miR-200c and miR-186. Moreover, miRNA inhibition resulted in the generation of a mechanism of resistance of ovarian cancer cells to the applied cisplatin-based treatment [178,185]. Chemoresistance leads to an increased pool of ovarian cancer stem cells. Resistin shows strong expression in ovarian cancer tissues and has been associated with a poor prognosis among patients diagnosed with ovarian cancer. Previous results suggest that due to resistin’s key role in EOC proliferation and migration, it may be useful as a target for ovarian cancer treatment [185].

### 7.2. Resistin in Endometrial Cancer

The latest available studies show that elevated resistin levels correlate with endometrial cancer stimulation, development and invasion, which is associated with advanced cancer stage and a worse prognosis for the patient [41,173,178,187,188]. Nergiz Avcıoğlu et al. [189] reported that resistin levels >0.27 ng/mL increased the risk of endometrial cancer by as much as 5.6 times. The pathomechanism of endometrial cancer development is associated with the activation of multiple signaling pathways, mainly the MAP kinase pathway, resulting in increased synthesis of P-selectin and fractalkine in endothelial cells [41,187,190]. In addition, resistin during ongoing inflammation increases the synthesis of pro-inflammatory factors, particularly TNF-α, IL-1, IL-6 and IL-12. It has also been proven that inflammatory markers can increase resistin levels [190].

The source of the occurrence of numerous diseases, especially cancer, is genetic disorders. SNPs, particularly the polymorphisms of the resistin gene 420 G > C and the 62 G > A gene, are believed to play an important role in the development of endometrial cancer. A study by Ozgor et al. [190] found that the 420 gene polymorphism positively correlated with the development of endometrial cancer. In contrast, interestingly, the 62 gene polymorphism showed a protective effect in the development of EC, despite being involved in the pathogenesis of PCOS.

## 8. Apelin

Initially, the cDNA for the orphan receptor—angiotensin domain type 1 receptor-associated protein (APJ) G-protein-coupled receptor—was cloned in 1993. However, it was not until 1998 that a ligand for this receptor, apelin (APLN), encoded by the *APLN* gene, was discovered, while in 2013, a second ligand for the APJ receptor, apelin ELA (Elabela) encoded by APELA, was found [191,192,193,194]. The apelin gene, *APLN*, is located on chromosome 10 (Xq 25–26) and encodes a 77 amino acid peptide called pre-proapelin, which undergoes N-terminal truncation by endopeptidase resulting in proapelin-55, which undergoes further truncation, resulting in the apelin family of proteins. This group includes apelin-12, -13, -16, -17, -19, and -36. The resulting apelins differ in the length of the protein chain. Shorter forms, e.g., apelin-13, are characterized by a much stronger degree of biological activity compared to longer forms, i.e., apelin-36. Hence the longer forms are converted into shorter forms. Each isoform has a C-terminal region through which binding to the APJ receptor is possible [191,192,193,195,196,197,198].

The gene for the second ligand is located on the 4th chromosome. It encodes a precursor, pre-proprotein, from which a 22-amino acid signal peptide and a 32-amino acid proprotein, also called apella-32, are formed in the next step. The peptide is removed, while apela-21 and apela-11 are formed from apella-32 following a cleavage reaction by furin. APELA exhibits biological activity during the development of the organism [193,196,197]. Compared to apelin and APJ, APELA expression is less widespread [199].

The specific receptor for both ligands is angiotensin-like receptor 1 (APJ). It is encoded by the *APJ* gene, which is located on chromosome 11q12. The APJ receptor is made up of 380 amino acids. The APJ receptor is conjugated to the G protein and shows homology with the angiotensin II type 1 receptor but is unable to bind Ang II [200,201].

Apelin expression has been demonstrated in many tissues and organs, particularly in the brain, placenta, heart, lungs, pancreas, testes, prostate and adipose tissue [202]. It has also been found that apelin expression increases during the adipocyte differentiation process. In addition, apelin synthesis is controlled by various factors, which include growth hormone (GH), tumor necrosis factor (TNF-α) and insulin. Insulin increases apelin synthesis by acting on adipose tissue cells [203].

Physiologically, apelin fills an important role in regulating the cardiovascular system by controlling blood pressure and vascular blood flow [195,203]. It influences the process of angiogenesis, stimulating the proliferation of endothelial cells and the migration and formation of blood vessels. Apelin exhibits anti-inflammatory properties by inhibiting the release of inflammatory mediators and shows antioxidant activity by reducing the release of reactive oxygen species in adipose tissue. It plays an important role in endocrine metabolism, affecting the hypothalamus and pituitary gland [203]. Kurowska et al. [203] report that apelin administration results in an increase in the secretion of corticotropin-releasing hormone (CRH) and vasopressin, indicating an important role in water metabolism. It increases the release of adrenocorticotropic hormone while it inhibits the release of the thyrotropic hormone, prolactin, folliculotropic hormone and luteinizing hormone [195].

Increased apelin expression in white adipose tissue and elevated apelin serum levels have been demonstrated in obese subjects associated with hyperinsulinemia. In addition, Apelin has been shown to play an important role in the hypothalamic-pituitary-gonadal (HPG) axis, affecting reproductive function in women as well as in men.

**Figure 6:**
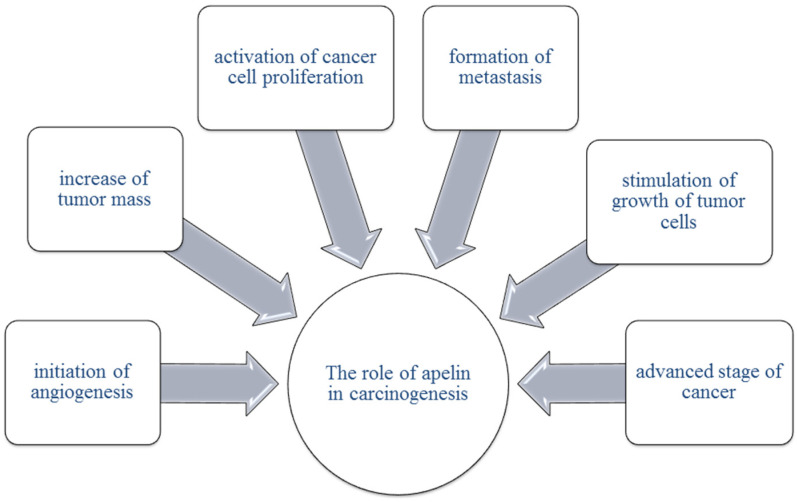
Potential role of apelin in carcinogenesis [200,206,207,208].

In recent years, there has been an increasing amount of data indicating a potential role for the apelin/APJ system in many cancers [200]. It is now known that the apelin/APJ system is involved in the development of many cancers, particularly ovarian, breast, lung, liver, prostate, and glioma [204,205]. The potential role of apelin in carcinogenesis is shown in Figure 6.

### 8.1. Apelin in Ovarian Cancer

Apelin plays an important role in the pathogenesis of gynecological diseases, mainly in PCOS [209], endometriosis [210] and ovarian cancer [208]. Elevated expression of this protein has been noted in ovarian cancer compared to physiological ovarian epithelial cells [203,211]. Studies conducted by Neelakantan et al. [211] using cell culture show that apelin affects survival, proliferation and metastasis formation by cancer cells. OVCAR-4 ovarian cancer cells treated with apelin showed enhanced growth and development. In addition, high expression of APJ favorably affects the process of metastasis in the peritoneal cavity. It has also been noted that apelin expression was higher in vivo than in vitro, and the development of cancer cells was more intense in vivo, which may be explained by the use of endogenous, adipocyte-derived apelin for cancer cell metabolism [211].

According to the study, the involvement of apelin in the processes of ovarian cancer development results in a shorter overall survival of patients by 14.7 months [211].

### 8.2. Apelin in Endometrial Cancer

Altinkaya et al. [212] were the first to show a particularly significant difference between serum apelin levels in patients with endometrial cancer compared to healthy women (215.1 ± 59.8 pg/mL vs. 177.3 ± 55.2 pg/mL). The authors also showed significantly higher levels of apelin in obese women with endometrial cancer (243.5 ± 49.2 pg/mL vs. 200.5 ± 52.7 pg/mL), which may indicate the involvement of apelin in the pathomechanism of endometrial cancer development. Moreover, apelin may be an independent risk indicator for endometrial cancer development in obese women [205,213].

## 9. Chemerin

Chemerin was first described in 1997 when a high expression of a then-unknown gene was noted while treating psoriatic lesions on the skin with tazarotene (a retinol derivative). Due to the nature of the induction of expression, this gene was designated *TIG2* (Tazarotene-Induced Gene 2). However, it was not until 2003 that the protein encoded by the *TIG2* gene, chemerin, was obtained as a ligand for the orphan receptor CMKLR1 (Chemerin Chemokine-Like Receptor 1) [214,215].

Initially, for a long time, nothing was known about the role of chemerin in the body, if only for the lack of sufficient research on the subject, while over time, more and more of its functions were discovered. As a result, it is now known that chemerin, in addition to chemotactic induction in dendritic cells, macrophages and NK cells [216], plays an important role in angiogenesis [217,218], adipogenesis [219,220], osteoblastogenesis [221], myogenesis [222], or glucose homeostasis [223]. In addition, chemerin plays an important role in inhibiting bacterial growth [223,224,225]. Physiologically, chemerin is expressed by many tissues and organs, such as in the lungs, heart, ovaries, kidneys and pancreas but is mainly produced by the liver and white adipose tissue [226,227,228,229]. As a rule, the concentration of chemerin in women and the elderly is higher than in men and young people, although physiologically, it ranges from 90 to 200 ng/mL [219].

Chemerin is also known as retinoic acid receptor-responsive protein (RARRES2). It is encoded by the *TIG2* gene located at position 7q36.1. The transcription and translation process results in a protein metabolically inactive product with a molecular weight of 16 kDa, consisting of 163 amino acids, called pre-prochemerin. The enzymatic removal by elastase and cathepsin G of the twenty terminal amino acids at the amino end produces pro-chemerin, secreted into the bloodstream and characterized by low biological activity. Prochemerin, on the other hand, undergoes further enzymatic processing and, as a result of the cleavage process at the carboxyl end by serine, cysteine and carboxypeptidase proteases, chemotactically active isoforms are formed, with chemerin 157 being the most active isoform. The enzymatic cleavage reaction can occur directly or sequentially [198,216,219,224,230,231,232,233,234,235,236,237,238].

The biological activity of chemokine isoforms is enabled by the existence of specific receptors, which include chemokine receptor 1 (also called chemokine-like receptor 1—CMKLR1), chemokine receptor 2—GPR1 (G Protein-Coupled Receptor 1), coupled to G protein, and chemokine-like receptor 2—CCRL2 (C-C Motif Chemokine Receptor Like 2) [227,236,238,239]. The CMKLR1 receptor is expressed mainly on the surface of monocytes, macrophages, Natural Killer cells (NK) and plasmocytoid dendritic cells, and its strong expression is found in adipose tissue. Activation of the receptor affects the mobilization of intracellular calcium and activation or inhibition of MAPK, ERK1/2, P38, PKB, AMPK and NFkB. The GPR1 receptor is not located on the surface of immune cells. Still, it is expressed by cells of the nervous system, mainly the hippocampus and U7 cells (glioblastomas), and unlike CMKLR1, it does not affect calcium mobilization. In contrast, the CCRL2 receptor is strongly expressed in lung endothelial cells and the liver. It controls the bioavailability of chemerin through its uptake and presentation to the CMKLR1 receptor found on surrounding cells [218,227,239,240,241]. De Henau et al. [242] showed that chemerin has an identical affinity for the CMKLR1 and GPR1 receptors while showing a lower affinity for the CCRL2 receptor.

It has been shown that an increase in chemerin concentration correlates with the degree of obesity and BMI. In addition, white adipose tissue deposits are believed to be the main source of excessive synthesis of this adipocytokine. The chemerin produced in this way may exhibit autocrine or paracrine effects [226,229]. An important role in the pathogenesis of obesity is played by the chemerin-CMKLR1 axis, which is involved in the process of differentiation of preadipocytes into adipocytes via AKT-mTOR and ERK signaling pathways [233].

**Figure 7:**
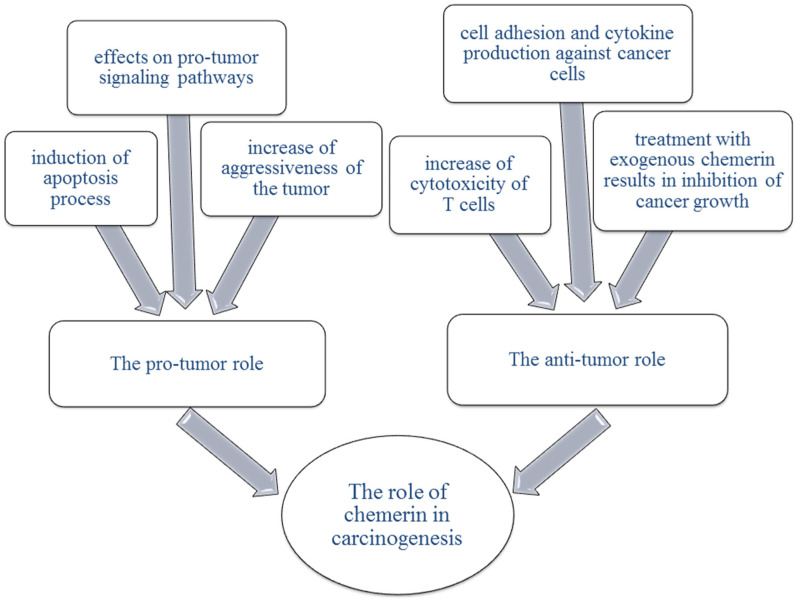
Potential role of chemerin in carcinogenesis [215,232,243,244,245,246,247,248].

Chemerin has a significant function in cancer development, yet its role in carcinogenesis is poorly understood. It turns out that the concentration of chemerin can be decreased or increased during the development of many cancers and can show both protective and promoting effects on the development of carcinogenesis [232,243,244]. The potential role of chemerin in carcinogenesis is shown in Figure 7.

### 9.1. Chemerin in Ovarian Cancer

Chemerin plays an important role during the development of ovarian cancer. Unfortunately, there is little information available on this subject. Still, among the available data, it is known that the concentration of chemerin in the ascitic fluid from patients with ovarian cancer is strongly elevated [229,249,250]. Gao et al. [249] report that the concentration of chemerin is significantly higher compared to the serum concentration. In addition, they showed that chemerin expression in ovarian cancer tissue was significantly higher than the expression of this adipokine in non-cancerous tissue. It has also been found that ascites is most often accompanied during the development of ovarian cancer, which promotes the adhesion of ovarian cancer cells. Moreover, adipocytes are capable of synthesizing IL-6, IL-8 and CCL2, which affect the process of metastasis formation. Hoffmann et al. [251], based on their study, concluded that the use of exogenous chemerin has no effect on both ovarian cancer cells and physiological ovarian cells. In contrast, Schmitt et al. [252] noted that chemerin in vitro reduced the number of cells in the OVCAR-3 line. They also noted that chemerin affected the activation of Interferon alpha (IFNα) response genes, mainly Interferon alpha-inducible protein 27 (IFI27), 2′-5′-Oligoadenylate Synthetase 1 (OAS1), Interferon Induced Protein With Tetratricopeptide Repeats 1 (IFIT1) and Interferon Regulatory Factor 9 (IRF9), and thus affected IFNα activity. The analysis showed a more than fourfold increase in the concentration of IFNα in the culture medium, which may indicate the effect of chemerin on IFNα. The authors emphasize that their study highlights for the first time the anti-tumor role of chemerin in ovarian cancer, where the mediator of this response is IFNα.

### 9.2. Chemerin in Endometrial Cancer

The role of chemerin in the pathomechanism of endometrial cancer development has still not been clarified. It has been suggested that chemerin plays an important role in reproductive functions. Chemerin expression has been found in the uterus, ovaries and placenta [253]. On the other hand, chemerin has been shown to be involved in the development of polycystic ovary syndrome (PCOS) [254]. Guzel et al. [255] showed significantly higher serum levels of chemerin in obese women diagnosed with PCOS compared to obese women without PCOS (8.98 ng/mL vs. 7.02 ng/mL). It is widely known that PCOS may be a predisposing factor in the development of endometrial cancer, which requires further research [256].

## 10. Omentin

Omentin (ITLN) is an adipokine composed of 313 amino acids with a molecular weight of 35 kDa [257,258,259,260]. It was originally identified in 2005 in intestinal Paneth cells and endothelial cells as intestinal lactoferrin receptor, a galactofuranose-binding lectin, and was named intellectin-1 [258]. It is now known that omentin is synthesized not by mature adipocytes but by a population of cells referred to as the log-vascular fraction, which includes preadipocytes, macrophages, lymphocytes and endothelial cells [261,262]. In addition, omentin expression has been demonstrated in numerous tissues and organs, particularly in the heart, lungs and ovaries [263].

Omentin is encoded by two genes located side by side in the chromosomal region 1q22-q23 and encode omentin-1 (ITLN1) and omentin-2 (ITLN2), respectively. These two isoforms are highly homologous to each other and show as much as 83% similarity in amino acid composition. Among the human population, the predominant isoform in serum is omentin-1. Physiologically, its serum concentration is 100–800 ng/mL. It has also been noted that compared to other adipocytokines, omentin has a positive correlation with adiponectin and a negative correlation with leptin [198,258,261,263,264].

**Figure 8:**
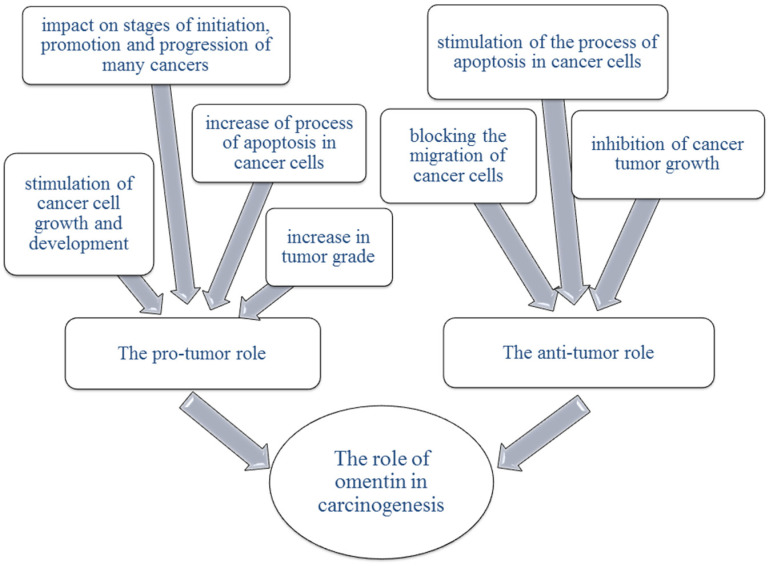
Potential role of omentin in carcinogenesis [268,269,270,271,272].

Omentin has been negatively correlated with the incidence of inflammation, obesity, diabetes and metabolic syndrome. Several studies have shown that overweight and obese individuals had reduced concentrations of omentin, and its expression in visceral adipose tissue was significantly reduced than in lean individuals. Moreover, omentin concentration is inversely correlated with BMI and percent body fat [260,261,265,266]. An increase in serum omentin levels correlated with a decrease in serum CRP levels and suppression of TNF-α function, confirming the link between ITLN and inflammation [267]. In addition, Escote et al. [261] noted that omentin-1 shows a gender dimorphism, as higher levels were observed in women than in men. It has also been shown that omentin interacting with paracrine and endocrine has an important function in glucose metabolism and insulin sensitivity, and omentin concentrations can also interact with glucose and insulin concentrations. Individuals with impaired glucose metabolism show reduced omentin-1 concentrations, which can lead to the development of metabolic syndrome, type 2 diabetes, and obesity and can lead to the initiation of the process of carcinogenesis [44,154,265]. The potential role of omentin in carcinogenesis is shown in Figure 8.

### 10.1. Omentin in Ovarian Cancer

In ovarian cancer, omentin levels are reduced in patients diagnosed with neoplasia compared to patients with benign gynecological lesions and in healthy women, which may confirm that ovarian cancer cells are capable of downregulating this adipocytokine [268,271,272]. Moreover, as indicated by Au-Yeung et al. [271], ovarian cancer cells can suppress ITLN1 expression in mesothelial cells in visceral adipose tissue for cancer cell proliferation and metastasis formation. On the other hand, it has been shown that adipocytes in the tumor microenvironment via omentin are capable of insulin-dependent glucose uptake. The corollary of this process is a local reduction in the glucose required by cancer cells for cellular metabolism, resulting in the inhibition of tumor growth.

### 10.2. Omentin in Endometrial Cancer

In their study, Cymbulak-Płoska et al. [273] showed that the concentration of omentin-1 during the development of endometrial cancer is significantly reduced compared to physiological serum levels. In addition, the authors showed a correlation between the concentration of omentin-1 and the stage of endometrial cancer. Meanwhile, others have studied the relationship between certain adipocytokines in treating endometrial cancer. For example, Soliman et al. [274] showed that omentin-1 levels decreased significantly after metformin treatment compared to before treatment. Yates et al. [275] obtained similar results in a later study. In addition, while analyzing the effect of omentin during endometrial cancer treatment, they noted that metformin has an inhibitory effect on the PI3K/AKT signaling pathway and reduces serum omentin levels. The authors emphasize that metformin causes AMP activation and phosphorylation of Live kinase B1 (LKB1) and thus affects the inhibition of the PI3K/AKT/mTOR pathway. This pathway is a commonly activated element in obesity-related endometrial cancer.

## 11. Vaspin

Vaspin was first isolated from visceral and subcutaneous white adipose tissue from obese OLETF (Otsuka Long-Evans Tokushima Fatty) rats burdened with type 2 diabetes [148,276,277]. It is a protein that in humans consists of 395 amino acids and has a molecular weight of 47 kDa [278]. It is encoded by the *SERPNA12* gene, located on the longer arm of chromosome 14 (14q32.13), includes five introns and six exons, and consists of 1245 nucleotides [279,280]. This adipokine has been shown to have 40% homology with α-1-antitrypsin and is included in the serine protease inhibitor (serpin) family [276,278]. In addition to adipose tissue, vaspin is synthesized by many other tissues and organs, particularly the stomach, liver, pancreas, heart, skin, small intestine and skeletal muscle. Serum vaspin concentrations ranged from 0.18 to 1.55 ng/mL [276,278,280]. Vaspin has also been shown to exhibit sexual dimorphism, as higher concentrations of vaspin are found in women than in men, which is explained by a probable relationship between this adipokine and steroid hormones [278,280,281]. In addition, vaspin exhibits a circadian rhythm closely related to food intake. The maximum concentration is observed in the early morning fasting period, while the minimum concentration is reached an average of two hours after a meal [281].

It is now known that there is a significant correlation between vaspin expression and BMI, body fat percentage and fasting glucose and insulin levels. On the other hand, low concentrations of vaspin have been reported in lean individuals and those who are regularly physically active, which may be related to the role of this adipokine in obesity [148,279,282]. In their analysis, Feng et al. [283] found that significantly higher levels of vaspin were found in obese individuals, confirming previous hypotheses.

Vaspin is clinically relevant in inflammation, exhibiting pro-inflammatory and anti-inflammatory characteristics [148,284]. As a pro-inflammatory factor, vaspin can be synthesized by adipose tissue-derived macrophages and inhibit macrophage apoptosis induced by several stress factors. In addition, it increases the secretion of IL-6, which enhances STAT3 activation [148]. Furthermore, Vaspin mediates the GRP78/MTJ-1 (Glucose-Regulated Protein 78/DnaJ-like 1 protein) signaling pathway, where it stimulates NF-κβ activation and pro-inflammatory cytokine synthesis [198]. Moreover, it has been shown that there is a significant relationship between vaspin and hs-CRP [284]. On the other hand, vaspin affected the inhibition of the expression of pro-inflammatory adipocytokines, mainly leptin, resistin and TNF-α, contributing to the suppression of inflammation [277].

To date, a specific receptor for vaspin has not been identified, but it has also been speculated that vaspin may be a ligand for the GRP78 receptor complex [281]. This receptor, through activation of multiple signaling pathways, plays a significant role in the control of numerous processes in the body, mainly in reproduction, tumor proliferation, neurological disorders or drug resistance [278].

**Figure 9:**
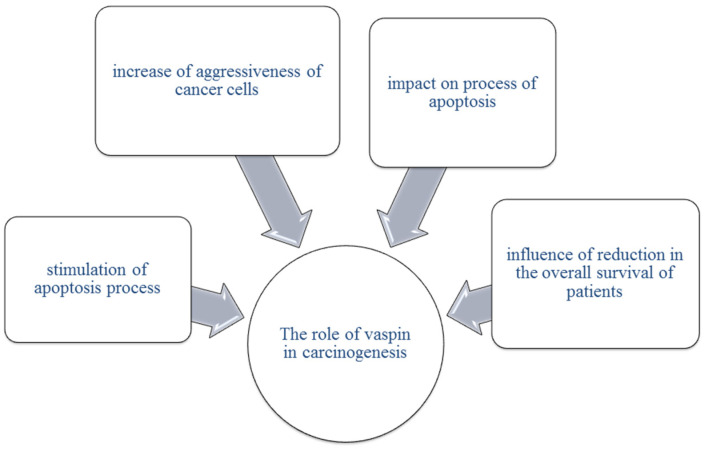
Potential role of vaspin in carcinogenesis [44,285,287].

Analyses conducted to date have indicated that vaspin plays a potential role in the carcinogenesis of many cancers, particularly colorectal cancer, hepatocellular carcinoma and breast cancer [148,285,286]. The potential role of vaspin in carcinogenesis is shown in Figure 9.

### 11.1. Vaspin in Ovarian Cancer

Kurowska et al. [203,278,280], in numerous studies conducted on porcine models, have shown that vaspin plays a key role in regulating fertility. By acting on the process of angiogenesis, vaspin stimulates the formation of a network of capillaries that supply luteal cells with nutrients, hormones and cholesterol so that ovarian endocrine function proceeds efficiently. The mechanism of angiogenesis stimulation is based on increased mRNA expression of pro-angiogenic factors (VEGFA, FGF2 and ANGPT1). On the other hand, vaspin inhibits apoptosis and stimulates luteal cell proliferation. The proapoptotic activity of vaspin is associated with a reduction in the activity of caspase 3 and 7, which play an important role in the process of apoptosis. In addition, there is an increase in the ratio of BCL2 to BAX, resulting in a reduction in the occurrence of apoptosis. The authors also noted that vaspin increases Proliferating Cell Nuclear Antigen (PCNA), a marker of proliferation, and cyclin A, which is responsible for the normal course of the cell cycle.

As the authors point out, maintaining homeostasis between apoptosis and proliferation is necessary to maintain a normal degree of fertility. The occurrence of disorders in the process of apoptosis and proliferation results in ovarian dysfunction, mainly infertility and the initiation of the process of carcinogenesis [203,278,280,288,289,290,291,292].

### 11.2. Vaspin in Endometrial Cancer

Currently, few studies are available on the role of vaspin in endometrial cancer. Nevertheless, available analyses indicate that vaspin levels are significantly reduced in the course of endometrial cancer [171,174,293,294]. Erdogan et al. [293] were the first to analyze vaspin levels in the course of endometrial cancer. The results obtained by the authors indicate that low serum levels of vaspin correlate with an increased risk of developing this cancer. Later, Cymbaluk-Płoska et al. [171], analyzing vaspin levels among a larger group of patients with endometrial cancer (n = 92), obtained identical results to the previous ones. Following this pattern, Kozłowski et al. [294] report that lower vaspin levels are found in patients whose cancerous lesions show a more advanced nature compared to a group of women whose cancer is less advanced. The authors report that their analysis does not indicate an association between vaspin levels and stage according to the FIGO classification.

The likely pathomechanism of endometrial cancer development associated with vaspin is related to stimulation of the insulin receptor (IR), as well as PI3K/AKT and MAPK/ERK signaling pathways. Interestingly, implementing PI3K and MEK inhibitors nullifies the anti-apoptotic nature and proliferative effects of vaspin, which may be useful in treating patients with this cancer [171,174]. The summary of the role of selected adipocytokines in carcinogenesis is shown in Table 1 below.

## 12. Conclusions

The studies conducted so far indicate the important role of adipocytokines in the process of carcinogenesis, which still seems to be not fully understood. The paper presents the role of selected adipocytokines, including leptin, adiponectin, visfatin, resistin, apelin, chemerin, omentin and vaspin, in the process of carcinogenesis based on available research. Interesting observations are provided by the analysis of adipocytokines carried out in ovarian and endometrial cancer, which may constitute a premise for further research in order to potentially use some of them in the development of new diagnostic and therapeutic schemes. Furthermore, since adipocytokines and their signaling pathways have been found to directly influence cancer cell proliferation, invasion and migration, these molecules have been indicated as potential targets for gynecological cancer treatment and prevention of progression. A promising strategy for harnessing the anti-carcinogenic effects of adipokines is the development of adipokine analogs or derivatives that mimic the biological functions of endogenous adipokines. However, further research is needed to fully understand and develop safe and effective strategies for their clinical use.

## Figures and Tables

**Table 1:** Comparison of the role of adipocytokines in different cancers. Adipocytokines in the pathomechanism of individual cancers manifest elevated or reduced concentrations with respect to reference values. The table considers this criterion, graphically assigning an arrow symbol: ↑—indicates elevated concentrations of adipocytokines, ↓—indicates decreased concentrations of adipocytokines, and the symbol ↑↓ indicates that both elevated and decreased concentrations of adipocytokines are involved in the mechanism of cancer pathogenesis.

	Other Kinds of Cancer		Ovarian Cancer		Endometrial Cancer	
**Leptin**	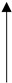	increase in tumor mass, facilitated metastatic ability, effect the process of angiogenesis, prevention of apoptosis of tumor cells [101,107,108,109,110]	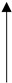	stimulation of cancer cell proliferation, inhibition of apoptosis, promotion of malignant cancer phenotypes, reduced survival rate [106,111,112]	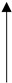	metastatic capacity, proliferation of cancer cells, induction of chemoresistance [30,41,105]
**Adiponectin**	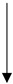	inhibition of cell cycle, blocking angiogenesis, induction of apoptosis [118,119,130,131,295]	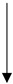	inhibition of angiogenesis, growth and proliferation, invasion, migration, formation of tumor metastases [62,108,114,127,133,134]	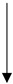	increase in cancer risk and stage [114,131,137]
**Visfatin**	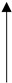	increase in risk of cancer development, shortened survival time, proliferation of cancer cells, metastasis formation [151,152,296]	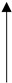	promotion of process of carcinogenesis, stimulation of process of angiogenesis, promotion of metastases formation [155,156,157,161,162]	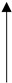	proliferative, proangiogenic effects, facilitated ability to form metastases [41,169,171]
**Resistin**	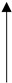	facilitation of cancer growth and development, increase in cancer stage, metastatic ability, increase in aggressiveness [53,54,94,162]	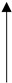	ability to promote and progress cancer, metastatic formation, angiogenesis promotion, induction of chemoresistance [53,178,184,185]	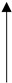	facilitation of growth and development of cancer, ability to metastasize, increase in stage of progression, decrease in survival rate [41,173,178,187,188]
**Apelin**	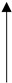	advanced stage of cancer, presence of metastases, induction of angiogenesis, poor prognosis [200,204,205,206]	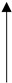	survivability, proliferation of cancer cells, metastasis formation, shortened survival time [203,208,211]	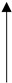	increase in cancer risk in women with obesity [205,212,213]
**Chemerin**	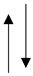	migration of immune system cells toward the tumor, intracellular signaling, facilitated angiogenesis [215,218,229,245,246,247,248,297]	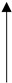	effect on cancer cell adhesion, metastatic capacity [229,249,250]		no data available
**Omentin**	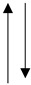	stimulation of cancer growth and development, metastasis formation, inhibition of proliferation, induction of apoptosis [268,269,270]	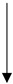	proliferation of tumor cells, metastasis formation [268,271,272]	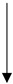	increase in stage of cancer [273,274,275]
**Vaspin**	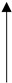	increase in degree of malignancy of cancer, decrease in survival time, formation of metastasis [44,287]	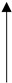	stimulation of process of angiogenesis, limiting the process of apoptosis [203,278,280]	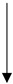	increase in risk of developing cancer, increase in stage of cancer [171,174,293,294]

## Data Availability

Not applicable.
